# Understanding late medieval farming practices: an interdisciplinary study on byre remains from the historical centre of Brussels (Belgium)

**DOI:** 10.1007/s12520-025-02248-w

**Published:** 2025-06-25

**Authors:** Yannick Devos, Cristiano Nicosia, Luc Vrydaghs, Jan M. A. van der Valk, Lien Speleers, Elena Marinova, Mona Court-Picon, Terry B. Ball, Christine Pümpin, Hugues Doutrelepont, Britt Claes

**Affiliations:** 1 Archaeology, Environmental Changes & Geo-Chemistry Research Group, Vrije Universiteit Brussel, Brussels, Belgium; 2 Università Di Padova, Padua, Italy; 3 Royal Belgian Institute of Natural Sciences, Brussels, Belgium; 4 Württemberg State Office for Cultural Heritage, Hemmenhofen, Germany; 5 Roots Asbl, Brussels, Belgium; 6 Brigham Young University, Provo, USA; 7 IPNA, University of Basel, Basel, Switzerland; 8 Royal Museums for Art and History, Brussels, Belgium

**Keywords:** Stable, Manure, Micromorphology, Archaeobotany, Palaeoparasitology, Medieval

## Abstract

During the preventive archaeological excavation in the cellar of the site of petite Rue des Bouchers in the historical centre of Brussels (Belgium), the waterlogged remains of a thirteenth century AD sunken byre (potstal) were discovered. The exceptional preservation instigated a multiproxy approach, including micro-archaeology, micromorphology, phytolith and endoparasite egg analysis on thin sections, palynology and the study of plant macroremains on the fill of the structure. Beyond permitting to detail the content and the multiple origins of the fill (including excremental waste and urines, fodder and bedding material, plaggen and/or soil sods, household and construction waste), this study also provides detailed insights into the foddering customs, hygienic conditions within the stable, and the health status of the animals kept. On a more general scale this study documented late medieval farming practices in Brussels, more specifically the need to collect substantial amounts of manure to add as fertilizer onto the cultivated poor sandy soils.

**Supplementary Information:**

The online version contains supplementary material available at 10.1007/s12520-025-02248-w.

## Introduction

Stables and stable manure are complex entities involving the accumulation of materials from different contexts, as well as different processes – both during the use of the structure, as well as post-depositional (Kenward & Hall 1997; Hall & Kenward 1998). Beyond informing us on the kind of animals that were kept, they can provide details on foddering practices, diet and the health status of the animals, waste disposal strategies, the environment of the site, etc. (see Dejmal et al. 2014; Banerjea et al. 2020b; Nicosia et al. 2022). On a more general scale they can also provide insights into farming practices, for instance the collection of manure and its application as a fertilizer. In order to come to a better understanding of such contexts, a holistic approach therefore seems appropriate. However, such studies – although very promising (see Albert et al. 2008; Dejmal et al. 2014; Macphail & Goldberg 2018; 354–368; Garcia-Suarez et al. 2018; Banerjea et al. 2020a & 2020b; Portillo et al. 2020; Alonso-Eguiluz et al. 2024) – remain rare. Most studies still tend to focus on one discipline or treat only one aspect. Soil physical and chemical analyses mostly deal with the detection of ancient stable structures (see Grabowski and Linderholm 2022 and references therein) and the manuring of fields (see Entwistle et al. 2000; Dercon et al. 2005), while botanical studies (for an overview, see Derremaux 2005), for instance, tend to focus on foddering practices and the study of excrements. The growing amount of micromorphological studies mostly deal with issues of structure identification and taphonomy (see Shahack-Gross 2017 and references therein). Interestingly, the study of individual coprolites often gives rise to a more interdisciplinary approach (for an overview on the latter see Linseele et al. 2013; Shillito et al. 2020), and especially promising in this respect are waterlogged sites, due to their excellent organic preservation (Kühn et al. 2013; Kenward and Hall 2012).

During the preventive archaeological excavation on the site of Petite Rue des Bouchers (BR229), in the historical centre of Brussels (Belgium), well-preserved remains of a late medieval stable fill were discovered (Claes 2018). An interdisciplinary study, including micro-archaeology, micromorphology, palynology and the study of plant macroremains, was performed in order to gain a better understanding of the structure, its content and use, the kind of animals kept, their feeding and health, and on a broader scale on late medieval farming practices. To fully exploit the potential of the thin sections, specific studies of phytoliths and endoparasite eggs were conducted. Whereas the study of phytoliths in thin sections has become a well-established method (see for instance Alonso-Eguiluz et al. 2025; Borderie et al. 2020; Devos and Vrydaghs 2023; Kovacs et al. 2020; Matthews 2010, and references therein), the study of endoparasite eggs in soil micromorphological thin sections is a quite young research field (see for instance Pichler et al. 2014; Pümpin et al. 2017), with many aspects that are still to be recognized and recorded systematically. One of the key advantages of this method – comparable to the analysis of the phytoliths – is the ability to gain insight into taphonomical processes and the repartition of the eggs within the sediment – the latter cannot be obtained through traditional palaeoparasitological approaches based on bulk sample analysis.

## Site and structure

Brussels is situated in the middle of the Belgian loess belt. However, in the historical town centre, these Aeolian deposits are rare, as along the steep slope and on the Brusselian plateau most soils developed directly on poor decalcified tertiary sands (see Schroyen 2003; Devos 2019), requiring substantial soil enrichment to obtain reasonable yields.

**Fig. 1:**
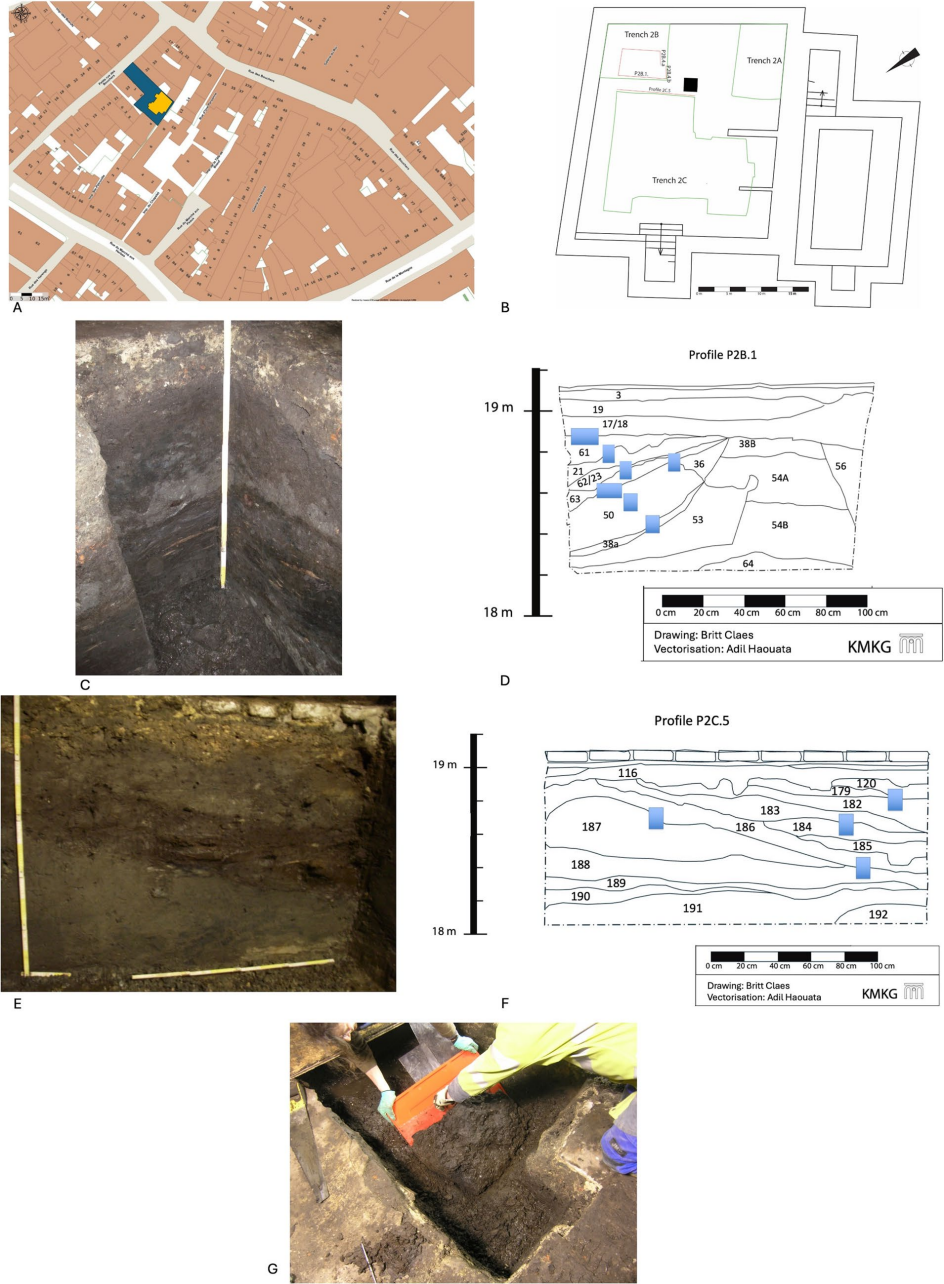
The studied site. **a **location of the site of Petite Rue des Bouchers (BR229). The site is indicated in blue, the cellars are indicated in orange; **b **detail of the cellar with indication of the excavated trenches and the location of the profiles were the fill of the sunken byre was observed; **c **photo of profile P2B.1; **d **drawing of profile P2B.1 (the micromorphological samples are indicated in blue); **e **photo of profile P2C.5; **f **drawing of profile P2C.5 (the micromorphological samples are indicated in blue); **g **photo of the bulk lifting of SU 23

The site of Petite Rue des Bouchers (**GPS** (Lat/Long) = 50,8477 4,3540) is situated within the alluvial valley of the Senne river at an altitude of approximately 20 m above sea level. In the cellar of the house n°29, remains of a stable fill were observed in profile P2B.4.a, P2B.4.b, P2B.1 in trench 2B and profile P2C.5 in trench 2 C (Figs. 1a-g)(Claes 2018). The structure, dated to the thirteenth century AD, has been dug within dark earth deposits dated to the eleventh-twelfth century AD. These dark earth deposits were identified as being of colluvial origin, possibly with some input of alluvial sediments (Devos et al. 2017). The upper part of the structure is truncated and covered with dark earth deposits dated to the fourteenth-fifteenth century AD. Due to the limited extent of the excavation trench within the cellar, the size of the structure could not be determined. Its fill is composed of a succession of thin layers of due to the prevailing waterlogged conditions rather well to well-preserved organic remains, and therefore particularly well-suited for a multi-proxy approach (Table 1).

**Table 1:** Overview of the analyses conducted on the different stratigraphical units (SU) of the fill of the structure. (X = studied)

SU	61	21	62/23	63	36	50	182	183	184	185	186
Micromorphology	X	X	X	X	X	X	X	X	X	X	X
Phytoliths	X	X	X	X	X	X					
Parasites	X	X	X		X	X					
Micro-archaeology			X								
Macrobotany						X					
Pollen			X								

## Material and methods

### Micromorphology (including the study of phytoliths and endoparasite eggs)

Samples for micromorphology have been taken from profiles P2B.1 and P2C.5 (Figs. 1d & 1f). One subsample of a coprolite has been taken from the bloc lifted from SU 23 (Fig. 1g). Thin sections (60 × 90 mm and 90 × 120 mm) were manufactured by the laboratory for Mineralogy and Petrography, Ghent University (Belgium), according to the guidelines of Benyarku and Stoops (2005). The thin sections were scanned with a flatbed scanner under normal light (NL), plane polarised light (PPL) and under crossed polarisers (XPL) following Arpin et al. (2002). Observations were made with petrological microscopes under plane polarised light (PPL), under crossed polarizers (XPL), and with oblique incident light (OIL) at magnifications of 25x, 100x, 200x, 400x, 500x, and 800x. Additionally, the thin sections were equally studied under UV and blue fluorescence at 200x, 400 × and 500 × magnification (Van Vliet-Lanoë 1991; Stoops 2017). The thin sections were described following the international nomenclature of Stoops (2021). The concept of “Soil Microfabric Type” (hereafter SMT) was adopted (see Macphail & Cruise 2001; Goldberg & Macphail 2006). Each SMT corresponds to a set of common micromorphological characteristics that can occur within different thin sections. They are labelled with progressive numbers. Variations within the same SMT are indicated by decimal numbers (for example SMT 1, SMT 1.1, SMT 1.2). Descriptions are recorded in I-GEOARCHRec (Lo Russo et al. 2023).

#### Phytoliths

Based on the micromorphological observations, a phytolith study was performed on 5 thin sections (ie. SU 61–21, SU 21–62, SU 62–63-36, SU 50 & SU 23). Phytolith nomenclature and inventories follow ICPN 2.0 (ICPT (Neumann et al.) 2019a & b). Observations were systematically conducted under PPL and XPL at magnifications of 100 × and 400 × to gain general and detailed views of the objects submitted to analysis.

Within the SMTs defined during the micromorphological study 20 to 40 observation fields were scanned systematically under PPL and XPL at a magnification of 400x.

In present case, the phytolith analysis consists of a four-step approach:

- a systematic recording of the distribution patterns of the phytoliths (for definitions see Vrydaghs et al. 2017);
- the recording of the orientation of the phytoliths within the articulated patterns (side, planar, transverse sensu ICPT 2.0. (ICPT (Neumann et al.) 2019b);
- The description of visibility, preservation and colour of the individual phytoliths following Vrydaghs & Devos (2020);
- The counting of the phytoliths within each distribution pattern to develop a semi-numerical approach; three categories were defined: ($\le$ 5; 5 < x $\le$ 10 and > 10).

Complementary, the thin section of a coprolite from stratigraphical unit (SU 23) was also extensively studied. This study primarily targeted articulated systems. The analysis of the systems was conducted following a four-step approach:

- The counting of the phytoliths within each distribution pattern to develop a semi-numerical approach; three categories were defined: ($\le$ 5; 5 < x $\le$ 10 and > 10);
- Their inventory according to ICPN 2.0 (ICPT (Neumann et al.) 2019a & b);
- The evaluation of the morphological diversity of the articulated systems (1, 2, 3 or more types);
- Whenever possible, the wave patterns of Elongate dentate/dendritic phytoliths following Rosen (1992) were recorded. Sufficiently large and appropriately oriented articulated Elongate dentate/dendritic phytoliths were further analysed following Vrydaghs et al. (2016) and Ball et al. (2016) to provide botanical identifications.

#### Endoparasite eggs

For the study of endoparasite eggs in Petite Rue des Bouchers four thin sections (i.e. SU 21–62, SU 62–63-36, SU 36–50 and SU 23) were analysed. The thin sections were scanned from bottom to top using a screening pattern of 0.5 cm^2^ in order to ensure a constant recording. Screening was carried out at magnifications of 100x, 200x, 400 × and 630 × with PPL and XPL. The identification relies on Thienpont et al. (1990) and Boch and Supperer (1983). To visualize the presence and distribution of helminth eggs within the sediment, their occurrence was mapped using Adobe Illustrator. Both identifiable and potential endoparasite eggs of each species were recorded using distinct color codes to represent them. A systematic morphometric analysis of the parasite eggs in the thin sections was not carried out, as the classic rehydration method (Bouchet et al. 2003) seemed more appropriate to obtain an accurate size ratio. However, some eggs were sporadically measured.

### Study of bulk samples

#### Micro-archaeology

Two bloc samples from SU 23 impregnated in a solution of water and ethanol have been dissected with a pair of tweezers under a binocular in order to get a better understanding of the 3D organisation of the sediment.

#### Plant macroremains (including wood and charcoal remains)

A subsample of 1L of sediment from SU50 was measured according to the water displacement method (Antolín et al. 2015) and subsequently wet-sieved using mesh sizes of 2 mm, 1 mm and 0.5 mm. Sieving residues were sorted, and carpological remains were identified and counted under a binocular microscope at 6.5–50 × magnification. Identifications were made using the reference collection of the Royal Belgian Institute of Natural Sciences, identification keys and seed atlases (e.g. Cappers et al. 2006; 2009). The nomenclature of the plant taxa follows Verloove & Van Rossum (2023). The 2 and 1 mm fractions were studied completely, while a representative part (38%) of the 0.5 mm residue was analysed. The counts from this fraction were extrapolated to the entire subsample volume.

Due to the scanning of the dried residue (10,5L) prepared for the archaeozoological and anthracological study, some additional species with large fruit stones could be added to the taxa list. From the sieving residues 150 random charcoal and 100 subfossil wood fragments were selected and identified under a high magnification reflected light microscope.

#### Palynology

Three samples from SU 23 have been studied. Two different extraction procedures were applied:

1: one set was processed following Faegri & Iversen (1989). Clay contamination was removed by ultrasonic sieving (5 μm mesh opening), although some pollen grains (like those of *Juniperus* species) could be lost during this procedure. Further, the acetolysis will reduce the finds of non-pollen-palynomorphs (NPP) and most of the intestinal parasite eggs.

2: one sample was processed according to the procedure applied at the Royal Belgian Institute for Natural Sciences for archaeological sediments. It is based on Bastin & Coûteaux (1966), Goeury & de Beaulieu (1979) and Nakagawa et al. (1998), involving dense media separation.

Observations were made using a transmitted light microscope at 400 × magnification. A minimum of 500 pollen grains was counted. Identification was based on identification keys (e.g. Moore et al. 1991; Beug 2004) and the reference collection of the Royal Belgian Institute of Natural Sciences. Nomenclature follows Lambinon et al. (1998). Non pollen palynomorphs (NPPs) were also recorded. Identification follows Van Geel (2001) and Van Geel et al. (2003). Additionally, for two of the three samples, the different *Cerealia* pollen were identified by application of phase contrast microscopy following Beug (2004). For this, 50 random *Cerealia* pollen grains were selected from 5 different slides per sample (10 grains per slide).

## Results

### Micro-archaeology:

The first sample originating from SU 23 is composed of intertwined flattened and compressed lenses, of which three main types have been recognised:

- A: the most common category is predominantly composed of fragmented leaves of grasses that were horizontally deposited and show different orientations. The leaves are embedded within a brown organic matrix.
- B: these lenses are dominated by grey silty sediments with humified organic material and clean sand sized quartz. Macroscopic organic remains are rare.
- C: this type is dominated by randomly distributed and unoriented plant macro remains (including small seeds).

Further, pupae of *Diptera* and fragments of *Coleoptera* were observed, as well as some horse- or donkeyhair.

The second sample is an ovoid, dense coprolite, composed of fragmented leaves of grasses randomly distributed within a matrix of largely decomposed plant material.

### Micromorphology

**Fig. 2:**
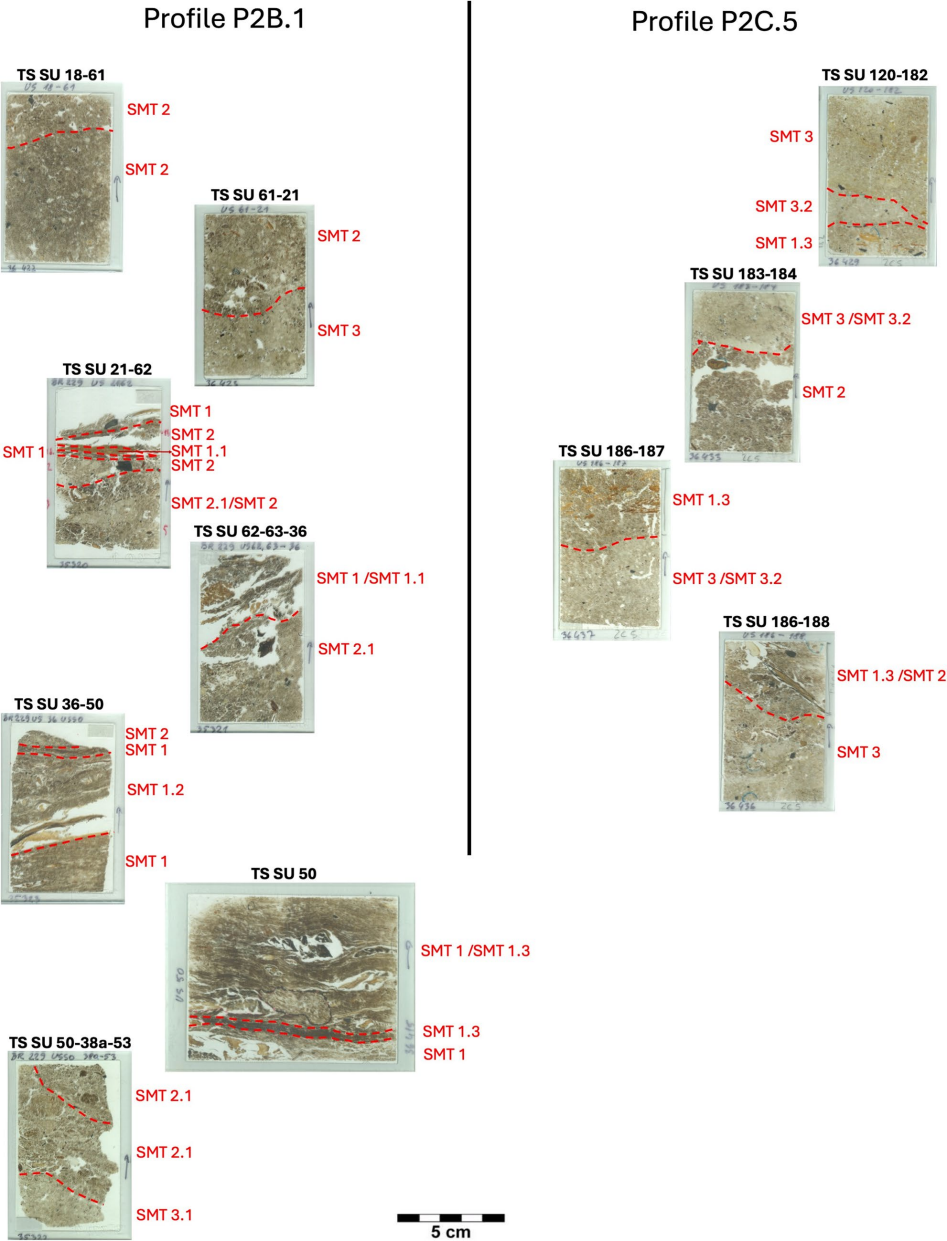
Scans of the thin sections (PPL) of the studied sequence with the location of the different Soil Microfabric Types (SMT) observed

**Fig. 3:**
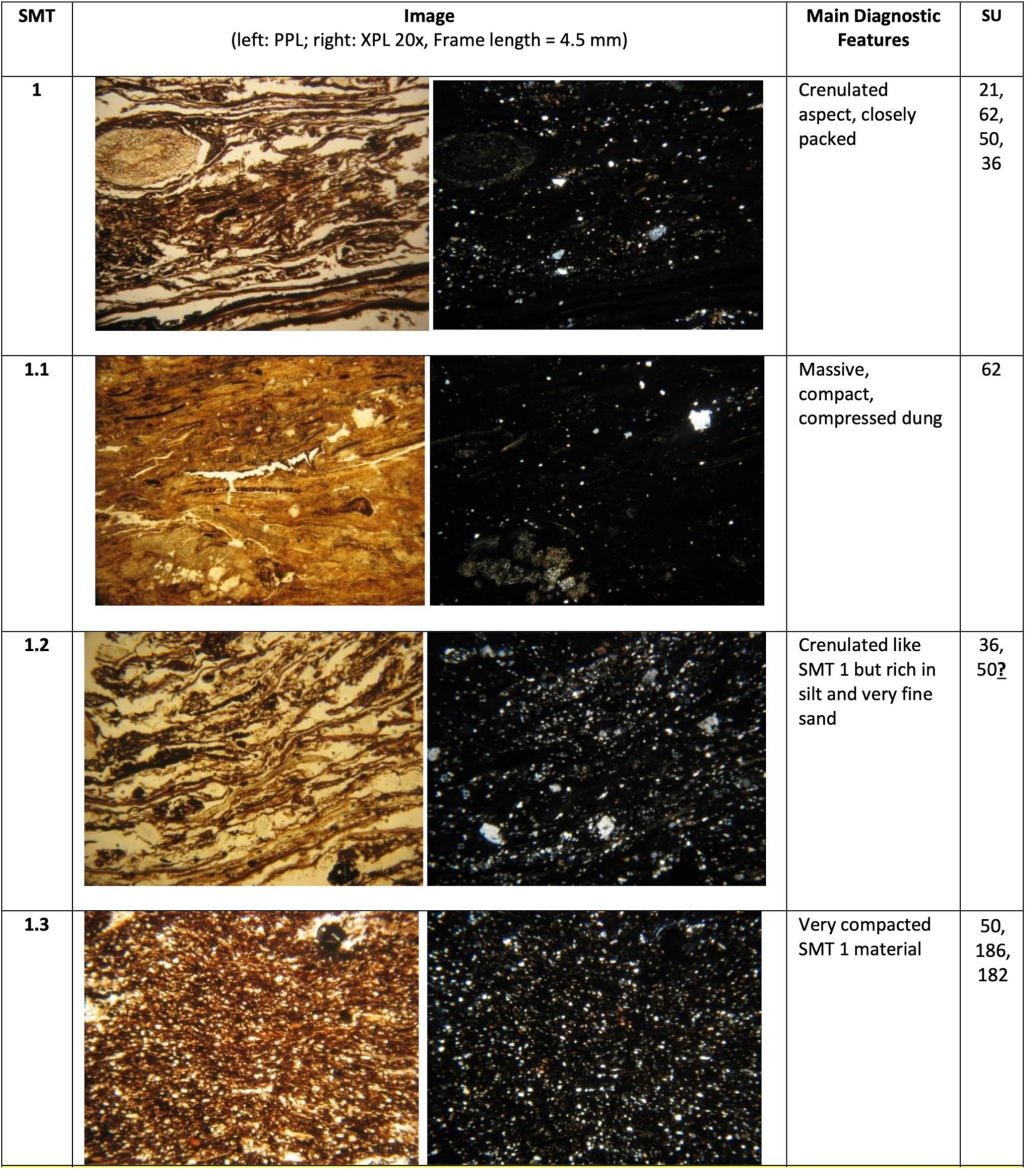
Main micromorphological characteristics of SMT1

**Fig. 4:**
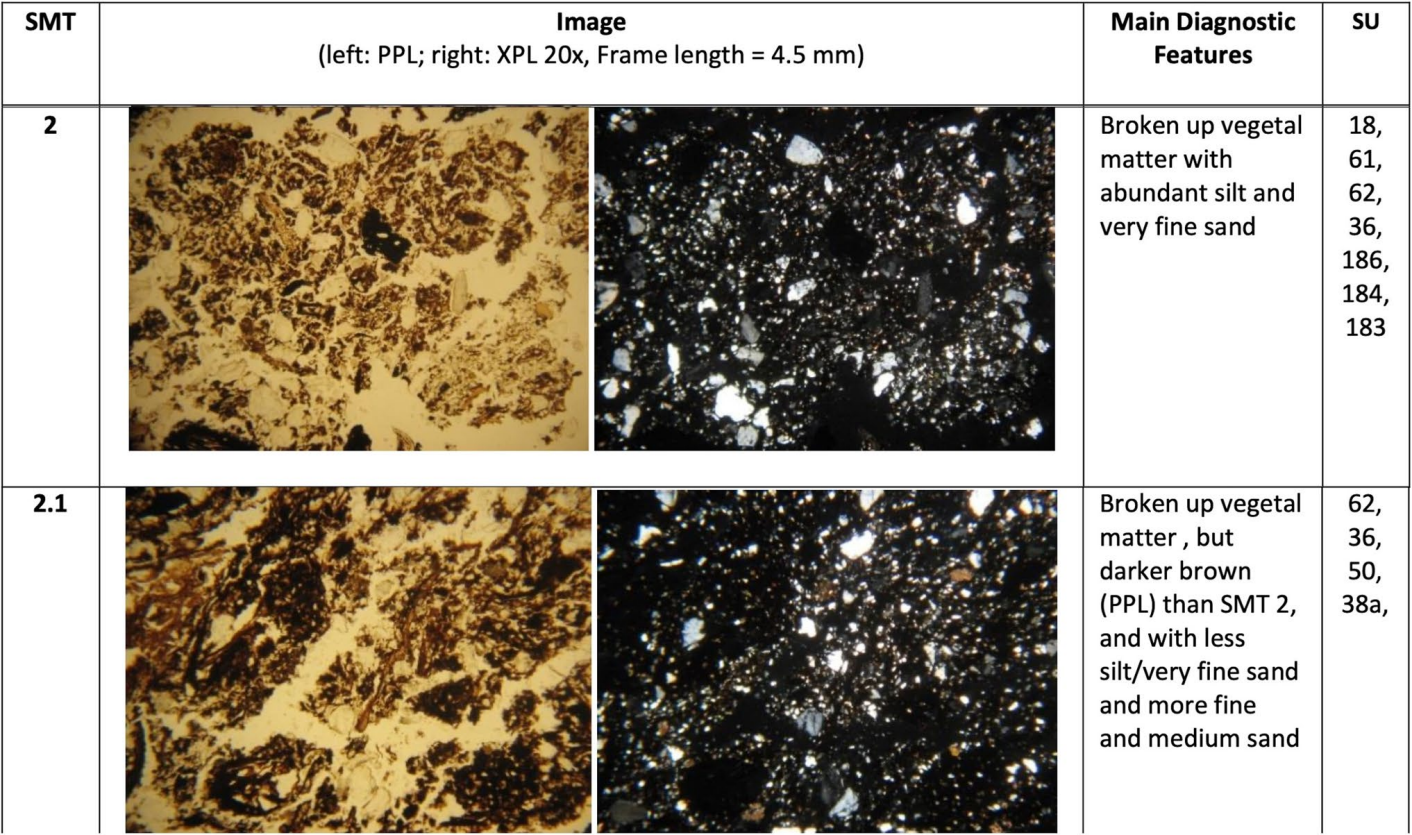
Main micromorphological characteristics of SMT2

**Fig. 5:**
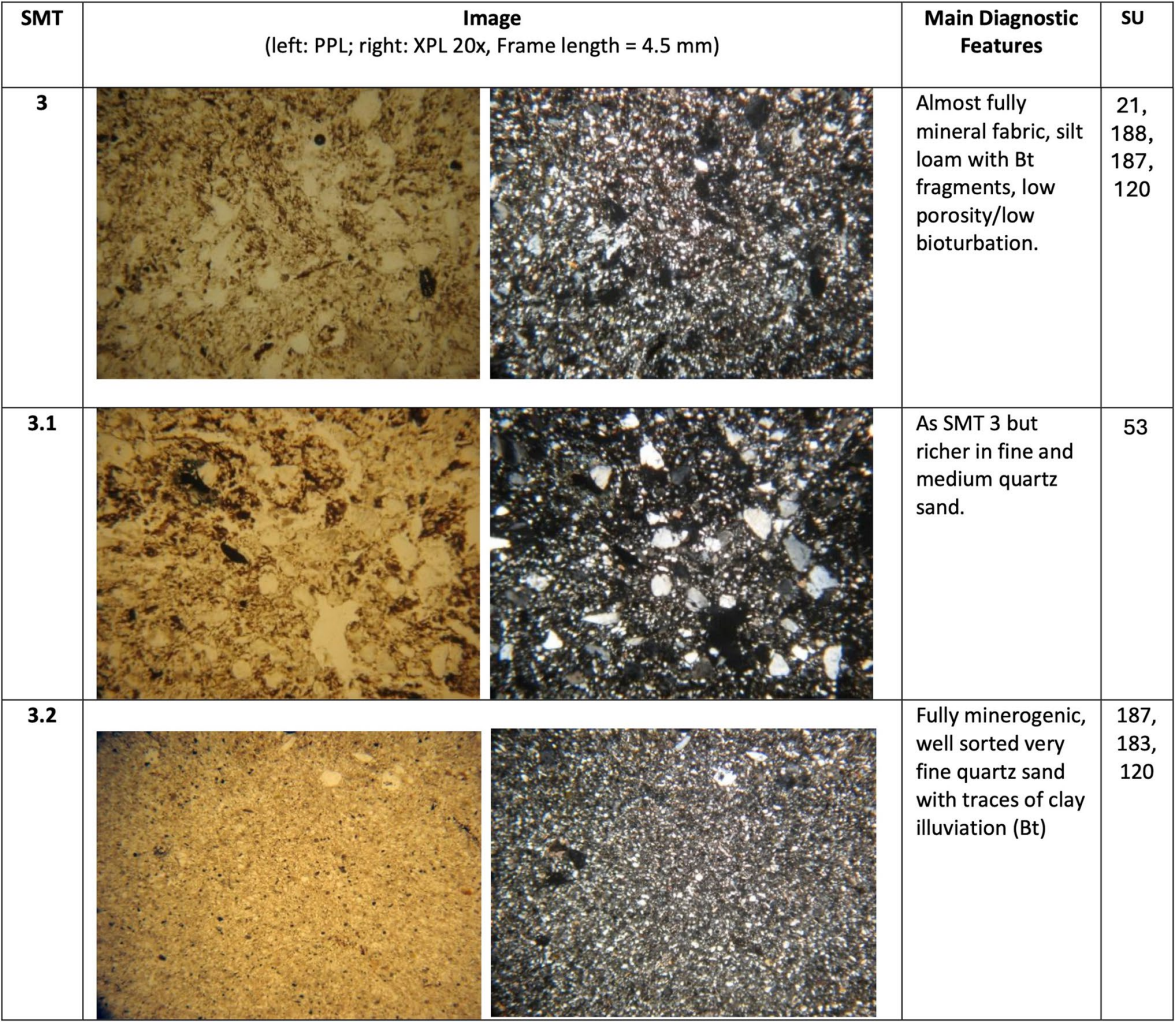
Main micromorphological characteristics of SMT3

The different SMT’s are presented in Fig. 2. The main characteristics of each SMT are summarised and illustrated in Figs. 3, 4 and 5. A summary of the opal of biogenic origin that has been observed in the SMT’s can be found in Table 2. Soil micromorphological descriptions of the SMT’s can be found in the supplementary information.

**Table 2:** Opal of biological origin

	Opal of biological origin			
	Phytoliths	Diatoms	Sponge spicules	Chrysophycean cysts
SMT 1	Very dominant	Rare		Rare
SMT 1.1	Very dominant	Rare		
SMT 1.2	Very dominant	Few	Rare	Few
SMT 1.3	Very dominant	Rare	Rare	Rare
SMT 2	Very dominant	Few	Rare	Few
SMT 2.1	Very dominant	Rare	Rare	Few
SMT 3	Very dominant	Few		Few
SMT 3.2	Very dominant			Few

Three main groups of SMT’s were observed:

SMT 1: this microfabric type includes all microstratigraphic units characterised by the presence of horizontally layered vegetal tissue fragments with a more or less crenulated aspect. Subtypes of SMT comprise:

SMT 1.1: composed of strongly compacted herbivore dung.

SMT 1.2: contains abundant mineral matter (silt and very fine sand).

SMT 1.3: characterised by a very dense appearance.

SMT2: this microfabric has been assigned to all microstratigraphic units composed of a mixture of aggregates or fragments of vegetal matter and of mineral components, without traces of internal layering.

SMT2.1 differs from SMT2 only for a coarser texture of the mineral part, comprising more medium sands.

SMT 3: this microfabric includes all microstratigraphic units consisting predominantly of mineral material, mostly with a silt loam texture. Organic matter is still abundant, but not as much as in SMT1 and SMT2. Frequent reworked fragments of Bt horizons were observed.

SMT3.1: differs from SMT3 in being richer in medium sands.

SMT3.2: aggregates of very well sorted very fine sand with occasional clay illuviation traces.

Throughout the studied sequence root and mesofaunal galleries are rare.

Main observed pedofeatures include vivianite nodules/crystal intergrowths (SMT 2, 2.1 & 3), phosphatic crystal intergrowths (SMT 2), pyrite framboids (SMT 1 & 2) and limpid clay coatings (SMT 3.2).

The preservation status of the plant remains varies from well-preserved remains still containing cellulose, over brownified to blackened and humified remains. In SMT 1.1 an important part of the plant remains also shows traces of mineralisation. SMT 3 and 3.2 are exceptions as most of the plant remains are humified.

#### Phytoliths

**Fig. 6:**
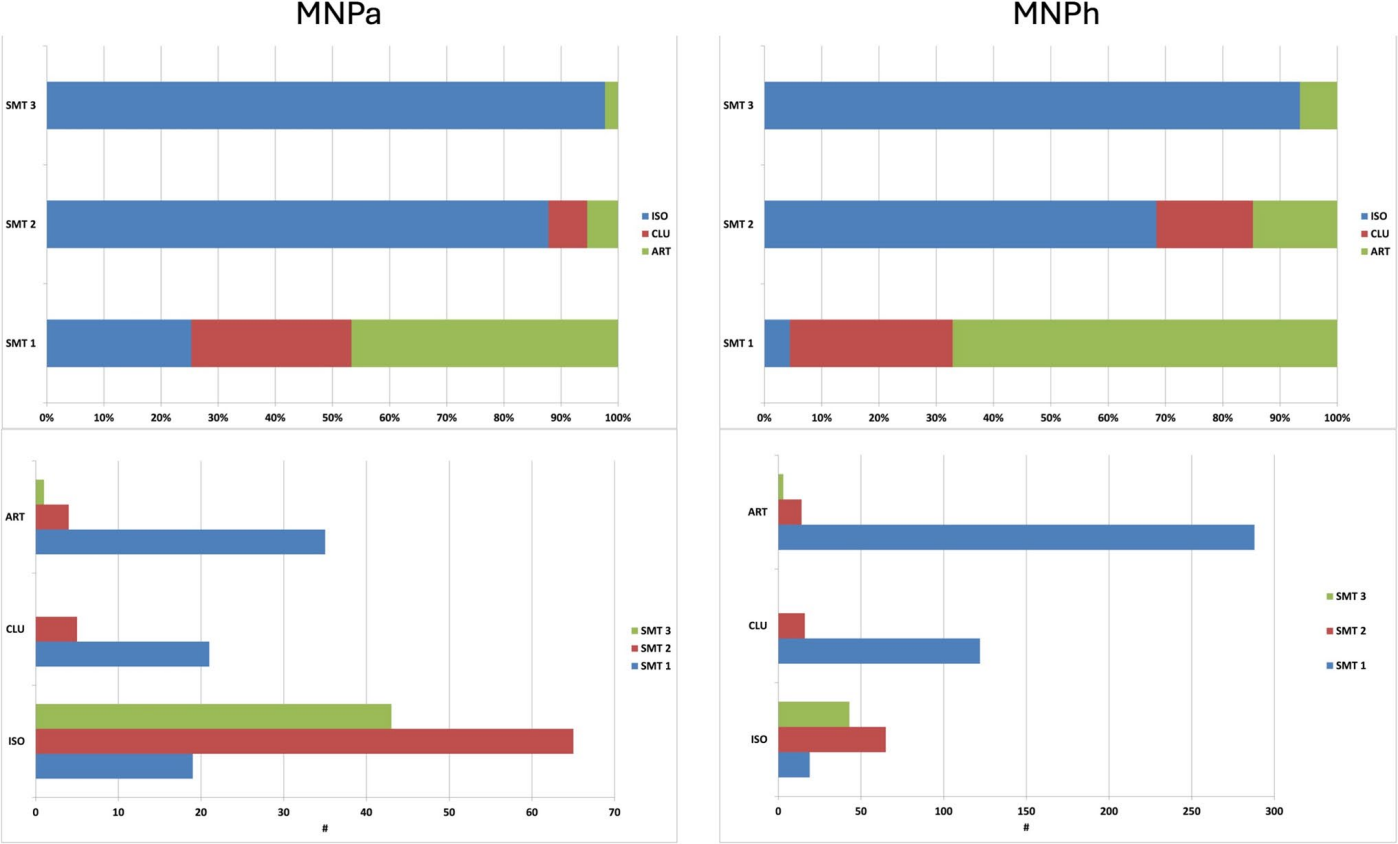
Minimal Number of Patterns (MNPa) and the corresponding Minimal Number of Phytoliths (MNPh) for each SMT based on the scanning of 40 fields. The upper histograms document the relative frequencies while the lower ones present their absolute values

Phytolith data are resumed in Tables 2, 3, 4 and 5 and Fig. 6.

**Table 3:** Anatomical attribution of 100 articulated systems from SU 23. The lack of attribution (Unknown) results either from the orientation of the system (side view) or the low resolution of the articulated phytoliths. One notes a clear prevalence of inflorescence bracts deriving from cereals, dominantly oat, indicating the animal was fed with byproducts of cereal processing

Botanical origin	#	Types	Wave pattern	
			Type	#
Inflorescence bract	71	ELO_DET/DEN	A	12
			B	13
			C	41
		PAP; RON		
Vegetative leaf/ Culm	17	ELO_ENT; ELO_SIN RON; TRZ; CRE ACU_BUL		
Unknown	12	ELO; ELO_ENT TRA		

Inflorescence bracts: ELO_DET/DEN: Elongate dentate/dendritic. Wave patterns: A: *Triticum* sp.; B: *Hordeum* sp.; C: *Avena* sp. (following Rosen 1992). PAP: Papillate; RON: Rondel. Vegetative leaf: ELO_ENT: Elongate entire; ELO_SIN: Elongate sinuate; RON: Rondel; TRZ: Trapezoid; CRE: Crenate; ACU_BUL: Acute bulbosus. Unknown: ELO: Elongate; ELO_ENT: Elongate entire; TRA: Tracheid.

**Table 4:** Morphological diversity within the 100 articulate systems analysed for SU 23

		#	Morphological diversity			
			Mono	Bi	Tri	Poly
SU 23; ART	< 5	10	7	2	1	
	5 < x < 10	19	12	6	1	
	> 10	71	32	24	9	6

Mono: articulated system involving one single type of phytoliths, most often Elongate dentate/dendritic; Bi: systems made of two types of phytoliths, mostly Elongate dentate/dendritic and Rondel; Tri: systems interlocking three types of phytoliths, Elongate dentate/dendritic, Rondel and another type of short cell (sensu Ellis (1979)); Poly: more than three types of phytoliths.

**Table 5:** Plant part (Inflorescence bract; Leaf/Stem and Unknown) attribution of the phytoliths recorded for SMT 1, 2 and 3. The lack of attribution (Unknown) results mostly from the low resolution and/or the orientation of the observed phytoliths

Inflorescence bract	SMT 1	ELO_DET; PAP; RON
	SMT 2	ELO_DET
	SMT 3	ELO_DET
Vegetative leaf; Culm	SMT 1	RON; BIL; BUL
	SMT 2	TRZ
	SMT 3	TRZ; POL
Unknown	SMT 1	ELO; ELO_ENT; TRA
	SMT 3	ELO_ENT

Inflorescences and vegetative leafs/culm are observed in all SMT’s. ELO_DET: Elongate dentate; PAP: Papillate; RON: Rondel; BIL: Bilobate; POL: Polylobate; BUL: bulliform; TEZ: Trapezoid; TRA: Tracheid.

The coprolite fragment of SU 23 is dominated by large randomly distributed and oriented articulated systems. These systems derive from vegetative leaves, culms and inflorescence bracts, part of them from cultivated cereals. The articulated systems deriving from inflorescence bracts of cultivated cereals draw A, B and C wave patterns, attributed to *Triticum* sp. (A), *Hordeum* sp. (B) and *Avena* sp. (C) (following Rosen 1992). As to the morphometrical analysis, it suggests to attribute the systems to *Triticum aestivum*, *T. dicoccon*, *Hordeum vulgare* and *Secale cereale*.

**Fig. 7:**
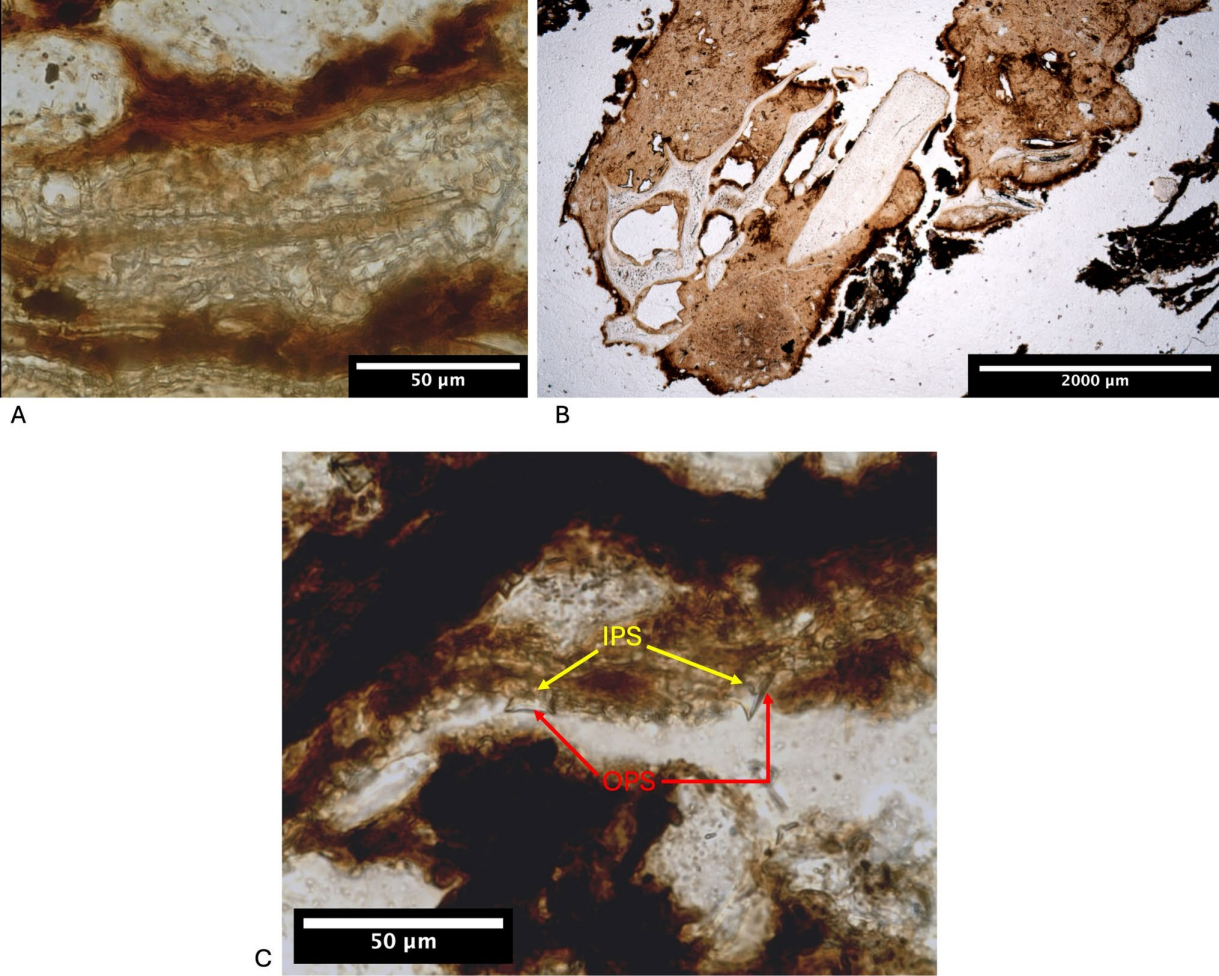
**A **microphotograph of densely packed phytoliths observed for SMT 1. While observed under XPL, latter material presents the isotropism typical for phytoliths (not presented here), under PPL, compaction of the material does not allow to discriminate any general outline; **B **microphotograph of an omnivorous coprolite (possibly human) observed in TS SU 62–63-36, SMT1/SMT1.1, PPL; **C **microphotograph of Rondel in an articulated system observed in SMT 1. Their Inner Periclinal Surface (IPS) is masked by amorphous organic matter while their Outer Periclinal Surface (OPS) is almost perfectly visible. As the IPS is the surface that faces towards the interior of the plant tissue (ICPT (Neuman et al.) 2019b), this observation indicates that the Rondel are oriented as they are when in anatomical position. As the OPS is almost perfectly visible, it also suggest a differential decomposition of the organic tissues (epidermal versus mesophyll), PPL

While SMT 3 and SMT 2 are largely dominated by isolated phytoliths, this is not the case for SMT1. This microfabric is instead dominated by parallel wavy oriented articulated systems. While within most of these articulated systems the phytoliths are observed in side view, others are observed in planar or cross section. All in-between orientations are also recorded (Devos and Vrydaghs 2023: Fig. 6). Such a combination of orientations hides a complex pattern of plant remains oriented along different axes. While at first glance one seems to observe an alternation of undulating micro-layers of opal and degraded organic matter (Shahack-Gross et al. 2003), detailed analysis demonstrates that the articulated systems are in this case part of the plant remains, as shown by the observation that the Rondel’s inner periclinal surfaces (IPS) of some systems are masked by (partly) decomposed organic matter: complete decomposition of the epidermis against humified mesophyll (Fig. 7c).

Further observations include broken phytoliths, densely packed phytoliths (Fig. 7a)(compaction) and phytoliths being slightly disarticulated.

#### Endoparasite eggs

**Fig. 8:**
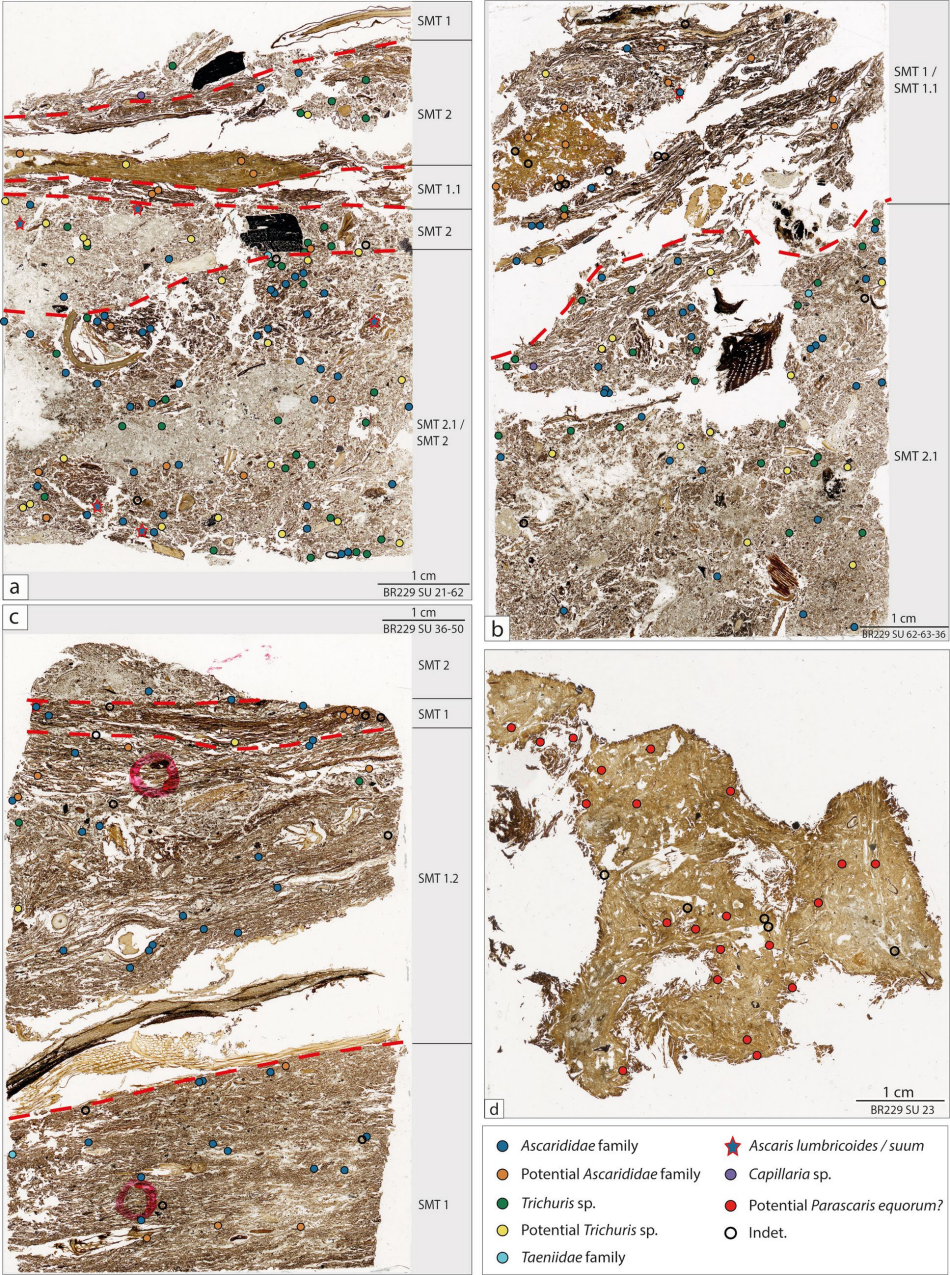
Scans of thin sections (**a **TS SU 21–62; **b **TS SU 62–63-36; **c **TS SU 36–50; **d **coprolite TS SU 23) with mapped endoparasite eggs

Table 6 summarises the number of eggs counted in the different thin sections. Figure 8 shows the distribution of the different types of intestinal parasite eggs within the studied thin sections.

**Table 6:** Endoparasite eggs identified in the analyzed thin sections."Indet."indicates components that cannot be clearly classified as endoparasite eggs, spores, or pollen

			Ascarididae	Potential Ascarididae	*Ascaris lumbricoides/Suum*	Potential* P. equorum?*	*Trichuris sp.*	Potential *Trichuris sp*.	*Capillaria sp.*	Taeniidae	Indet	Total eggs without indet
BR229	US 23		0	0	0	23	0	0	0	0	5	***23***
BR229	US 21/62	SMT1	0	0	0	0	1	0	1	0	0	***2***
		SMT2	2	3	0	0	4	2	0	0	0	***11***
		SMT1.1	0	2	0	0	0	0	0	0	0	***2***
		SMT2	3	1	2	0	6	6	0	1	1	***19***
		SMT2.1/SMT2	37	6	3	0	21	14	1	0	2	***82***
BR229	US62, 63–36	SMT1/SMT1.1	5	12	1	0	1	1	0	0	8	***20***
		SMT2.1	27	11	0	0	20	12	1	1	2	***72***
BR229	US 36 US50	SMT2	2	0	0	0	0	0	0	0	0	***2***
		SMT1	2	3	1	0	0	1	0	0	3	***7***
		SMT1.2	15	4	0	0	2	1	0	0	3	***22***
		SMT1	11	4	0	0	0	0	0	1	3	***16***
		***Total:***	***104***	***46***	***7***	***23***	***55***	***37***	***3***	***3***	***27***	***278***

In the thin section SU 21/62 (Fig. 8a) the helminth eggs are mainly distributed in the lower and the middle part of the thin sections (SMT 2.1/SMT 2 and SMT 2). Most of the ova are of the family of *Ascarididae*, concentrated in the organic rich part of the sediment.

Whereas overall in the thin section SU 62/63/36 (Fig. 8b), intestinal parasite eggs are homogeneously distributed, a concentration of *Trichuris* sp., rare in SMT 1/SMT 1.1, can clearly be localised in soil microfabric type SMT 2.1.

In the lowest studied thin section (SU 36/50) (figs. 8c), the most frequently observed eggs of intestinal parasites belong to the family of *Ascarididae*. These ova are homogeneously distributed throughout the sample and show no significant variation among the identified microfabric types (SMT 1, SMT 1.2 and SMT 2).

**Fig. 9:**
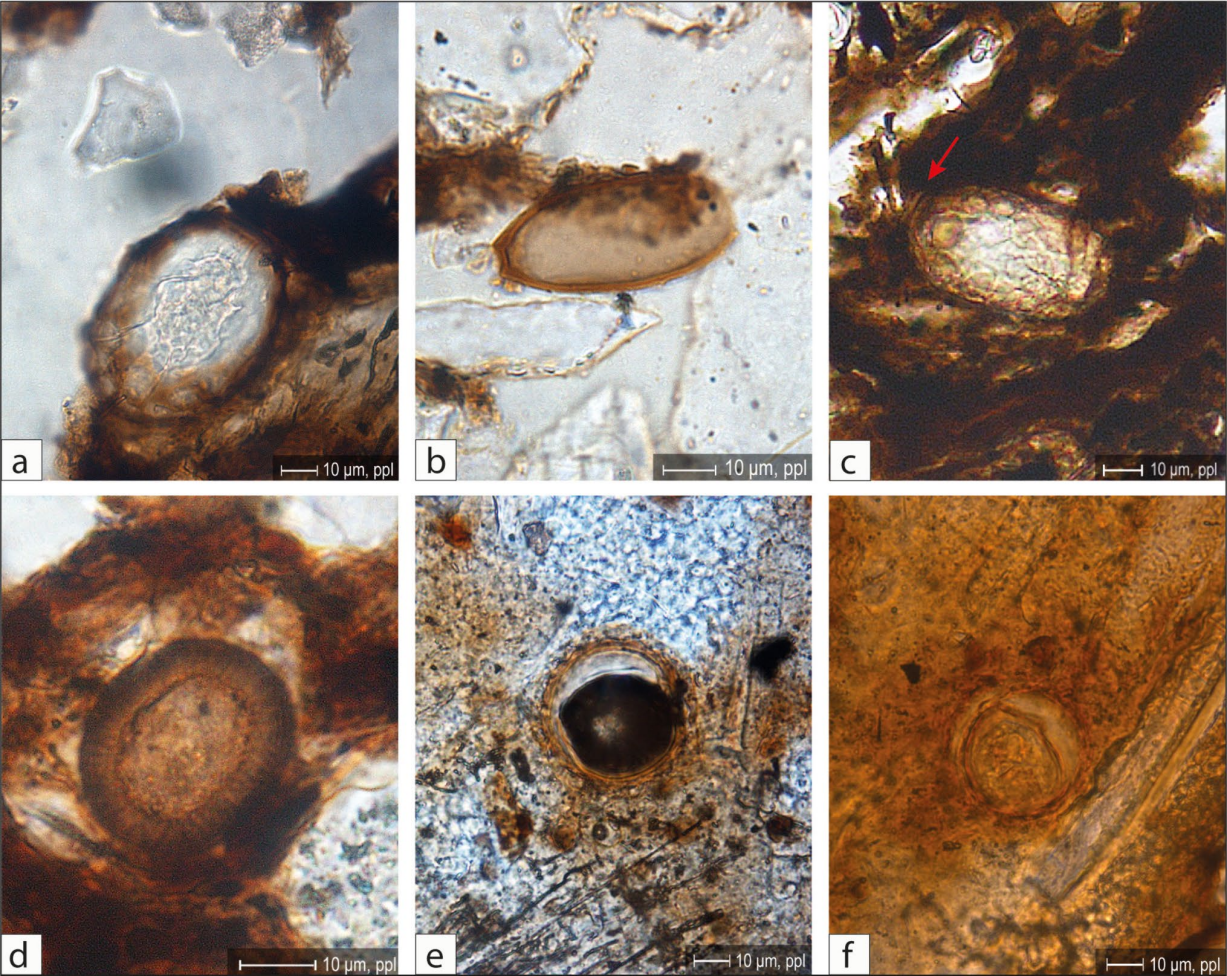
**a **Micropicture of an *Ascaris lumbricoides* or *A. suum* egg, parasitizing humans or pigs (PPL). The undulated shell, typical for these species, is partially shed due to taphonomicaly processes; **b **Micropicture of a *Trichuris* sp*.* egg with its typical lemon shape (PPL); **c **Micropicture of a *capillaria* sp. egg, identified by its lemon shape and distinct reticulated surface. The pole on the left side (red arrow) is visible, the right one is masked by the organic matrix (PPL); **d **Micropicture of a Taeniidae egg of a potential *Taenia* sp. (the secant cut resulting in a reduced diameter) or an *Ecchinococcus* sp. showing the characteristic radial striation of the shell (PPL); **e** & **f **Potential eggs of *Parascaris equorum* with a thick, finely punctuated shell. Taphonomic processes may have resulted in size reduction of the eggs, an observation requiring further investigation (PPL). (**a **TS SU 62–63-36; **b**—**d **TS SU 36–50; **e**–**f **TS SU 23)

In all three thin sections, the most common helminth eggs are of the *Ascarididae* and *Trichuridae* families. The determination of species was feasible in only a few cases due to the random cut of the thin section and the state of preservation of the eggs. In particular, the recognition of *Ascaris lumbricoides* or *A. suum* was possible (Fig. 9a; Table 6). *Trichuris* sp. is well represented (Fig. 9b), while eggs of *Capillaria* sp. (Fig. 9c) are observed in only small numbers. Eggs of tapeworm (*Taeniidae* family) are also present in limited quantities (Fig. 9d).

In the coprolite fragment of SU 23 the eggs of intestinal parasites are well spread in the matrix. The number of ova is rather low, and the specimen present are difficult to identify with certainty. One egg type has a shell morphology consistent with *Parascaris equorum* or *Toxocara vitulorum* (Figs. 9e-f). However, the observed sizes of these eggs are smaller than typical for these species.

### Plant macroremains *(including wood and charcoal remains)*

**Fig. 10:**
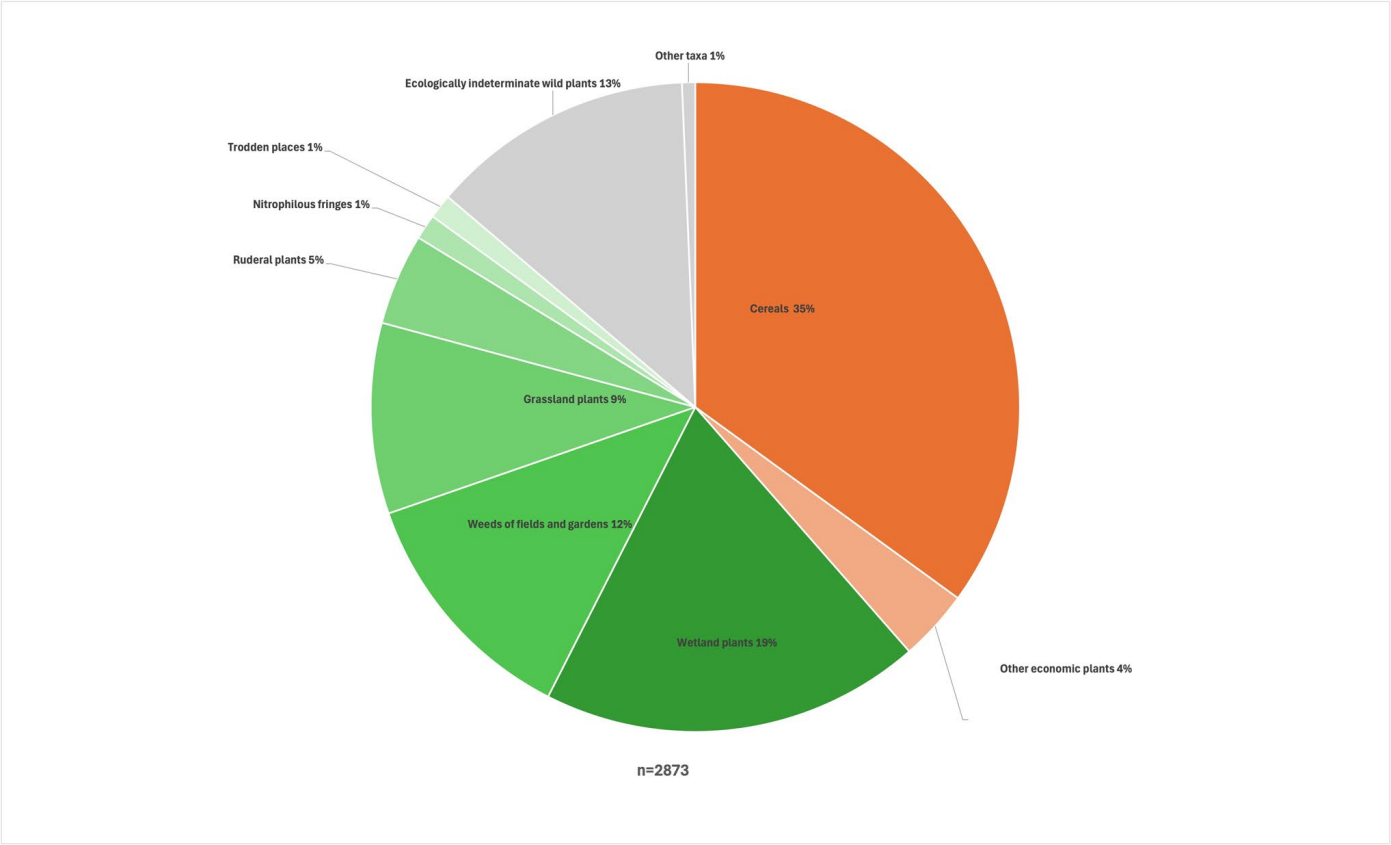
Proportions of the carpological remains in SU 50 (% of the total counted remains). For more details see Table 5

Table 7 and Fig. 10 summarize the carpological data.

**Table 7:** Results of the macrobotanical study of SU50

	#	% of total		#	% of total
Economic plants					
CEREALS			**GRASSLAND**		
*Avena* sp. (floret base)	380	13%	*Arenaria serpyllifolia*	3	0%
*Avena sativa* (floret base)	263	9%	*Carex leporina*	11	0%
*Secale cereale* (rachis internodium)	364	13%	*Cerastium arvense/fontanum*	93	3%
Cerealia Indet. (culm fr.)	+ + +	-	*Hypochaeris radicata*	5	0%
FRUITS AND NUTS			*Knautia arvensis*	2	0%
*Corylus avellana* (fr.)	+	-	*Medicago lupulina*	2	0%
*Fragaria vesca*	71	2%	*Leontodon autumnalis/hispidus*	32	1%
*Juglans regia* (fr.)	+	-	*Lychnis flos-cuculi*	13	0%
*Malus sylvestris*	1	0%	*Plantago lanceolata*	1	0%
*Prunus* cf.* avium*	14*	-	*Prunella vulgaris*	11	0%
*Prunus domestica*	2*	-	*Ranunculus acris/bulbosus/repens*	74	3%
*Prunus spinosa*	9*	-	*Stellaria graminea*	3	0%
*Prunus* sp.	+	-	*Trifolium pratense* (calyx)	23	1%
*Pyrus communis*	1	0%	**WETLAND**		
*Rubus fruticosus*	7	0%	*Bidens tripartita*	1	0%
*Rubus idaeus*	2	0%	*Bidens* sp.	4	0%
*Sambucus nigra*/*racemosa*	11	0%	*Carex panicea*	1	0%
*Vitis vinifera*	2	0%	*Eleocharis palustris*	55	2%
VEGETABLES		0%	*Glyceria fluitans*	5	
*Beta vulgaris*	1	0%	*Glyceria notata*	1	0%
*Daucus carota*	1	0%	*Glyceria* sp.	1	
*Pastinaca sativa*	1	0%	*Juncus bufonius*	3	0%
KITCHEN HERBS			*Lycopus europaeus*	1	0%
*Brassica nigra*	2	0%	*Mentha aquatica/arvensis*	48	2%
OIL AND FIBRE CROPS			*Oxybasis rubra*	162	6%
*Linum usitatissimum*	2	0%	*Persicaria hydropiper*	14	0%
			*Persicaria lapathifolia*	118	4%
Wild plants			*Ranunculus sceleratus*	45	2%
FIELDS AND GARDENS			*Rorippa sylvestris*	3	0%
*Agrostemma githago*	2	0%	*Rumex crispus*	39	1%
*Agrostemma githago* (fr.)	+ +	-	*Rumex crispus/obtusifolius*	33	1%
*Anchusa arvensis*	1	0%	*Salix* sp.	1	0%
*Anthemis cotula*	94	3%	*Salix* sp. (bud)	6	0%
*Centaurea cyanus*	5	0%	*Schoenoplectus tabernaemontani*	2	0%
*Chenopodium album*	18	1%	*Stachys* cf.* palustris*	2	0%
*Chenopodium polyspermum*	2	0%	**ECOLOGICALLY INDETERMINATE**		
*Echinochloa crus-galli*	88	3%	Asteraceae	15	1%
*Fallopia convolvulus*	2	0%	*Atriplex* sp.	28	1%
*Lamium purpureum*	4	0%	*Bromus* sp.	26	1%
*Misopates orontium*	21	1%	*Carex* sp.	4	0%
*Orlaya grandiflora*	1	0%	*Centaurea* sp. (fr.)	+	-
*Papaver argemone*	5	0%	Chenopodiaceae	180	6%
*Persicaria maculosa*	3	0%	*Myosotis* sp.	13	0%
*Raphanus raphanistrum* (fr.)	+ +	-	*Persicaria* sp.	0	0%
*Rumex acetosella*	47	2%	Poaceae	106	4%
*Scleranthus annuus*	8	0%	Poaceae (culm fr.)	0	0%
*Setaria pumila*	10	0%	*Ranunculus* sp.	0	0%
*Sinapis arvensis* (fruit fr.)	+	-	*Rumex* sp.	0	0%
*Sinapis arvensis* (seed)	5	0%	*Veronica* sp.	2	0%
*Solanum nigrum*	16	1%			
*Sonchus arvensis*	2	0%			
*Sonchus asper*	1	0%	**Other taxa**		
*Sonchus oleraceus*	3	0%	Brassicaceae (fr.)	+	-
*Spergula arvensis* var.* arvensis*	11	0%	Bryophyta (stem with leaves)	+	-
*Stellaria media*	1	0%	Fabaceae (ca.)	3	0%
*Thlaspi arvense*	1	0%	Fungi (sclerotium)	3	0%
RUDERAL PLACES			Rosaceae (thorn)	5	0%
*Chenopodiastrum murale*	54	2%	*Sinapis* sp./*Brassica* sp.	1	0%
*Conium maculatum*	32	1%	Indet. (bark, fr.)	+	-
*Linaria vulgaris*	4	0%	Indet. (dicotyl leaf, fr.)	+	-
*Reseda luteola*	8	0%	Indet. (flower)	1	0%
*Urtica urens*	33	1%	Indet. (seed/fruit, mi.)	5	0%
TRODDEN PLACES			Indet. (seed/fruit, mi.)	1	0%
*Plantago major*	12	0%	Indet. (roots, fr.)	+	-
*Polygonum aviculare*	23	1%	Indet (stem, fr.)	+	-
NITROPHILOUS FRINGES			Indet (twig, fr.)	+	-
*Galeopsis bifida/speciosa/tetrahit*	2	0%	Indet. (wood, fr.)	+ + +	-
*Lapsana communis*	2	0%	Indet. (wood, ca., fr.)	+ +	-
*Urtica dioica*	32	1%	**Other finds**		
			leather	+	-
			**TOTAL COUNTED REMAINS**	**2873**	

The remains listed are waterlogged seeds and fruits counted in the 1L subsample, unless otherwise specified (fr.: fragments, ca.: carbonised, mi.: mineralized, + : few, + + : moderate, + + + : many, *: remains observed in the dried residue of the larger bulk sample (10,5L)).

A very high concentration and diversity (108 taxa) of carpological remains is attested. Besides carpological remains, many wood fragments, as well as few to moderate amounts of bark, leaf fragments, mosses, stems, twigs, roots, charcoal and some fragments of leather were also observed in the sample. However, the sieving residue consisted largely of uncounted culm fragments of *Cerealia* (straw). The spectrum is characterised by an important fraction of cereal chaff including floret bases of *Avena* sp. and *Avena sativa* and rachis segments of *Secale cereale* (Fig. 10). Remains of other economic plants were found in small amounts (4% of the counted remains). These mainly include fruit pips, but also some nut scales, few seeds and fruits of vegetables, kitchen herbs and flax were found.

Remains of wild plants mainly include seeds and fruits of wetland vegetation, weeds of fields and gardens, grassland plants and ruderals. Smaller numbers of remains indicating trampled places and nitrophilous fringes were also found. Such an association of plants of different contexts is commonly found in stable manure (see Hall & Kenward 1998, Fig. 4).

#### Wood and charcoal

Table 7 summarizes the wood and charcoal data. A clear dominance of beech (*Fagus*) is observed in the charred assemblage. The sub-fossil wood is dominated by oak (*Quercus*). The uncharred wood also contained > 10% of bark fragments and in some cases twigs, partly of hazel, partly unidentifiable due to their small diameter (Table 8).

**Table 8:** Results of the study of charred and subfossil wood

stratigraphic units	SU 18		SU 50	
wood preservation type	charred	subfossil	charred	subfossil
total wood fragments	150	100	150	100
*Fagus*	81	7	107	4
*Quercus*	54	59	35	89
*Fraxinus*	7	11	3	
*Acer*		1		
*Corylus*		5		
*Alnus*		8	5	
*Salix*	8	5		5
*Sambucus*				2
*Cornus*		3		
Maloideae				
Prunoideae		1		
twig (indet.)	2	12	1	
bark fragment		9		19
indet	5	7	4	3
**other remains**				
dung fragments	+		+ +	
*Prunus* sp. (fruit stone)		1		1
*Corylus avellana* (nut shell)				1

(+ : few, + + : moderate, + + + : many).

### Palynology

**Fig. 11:**
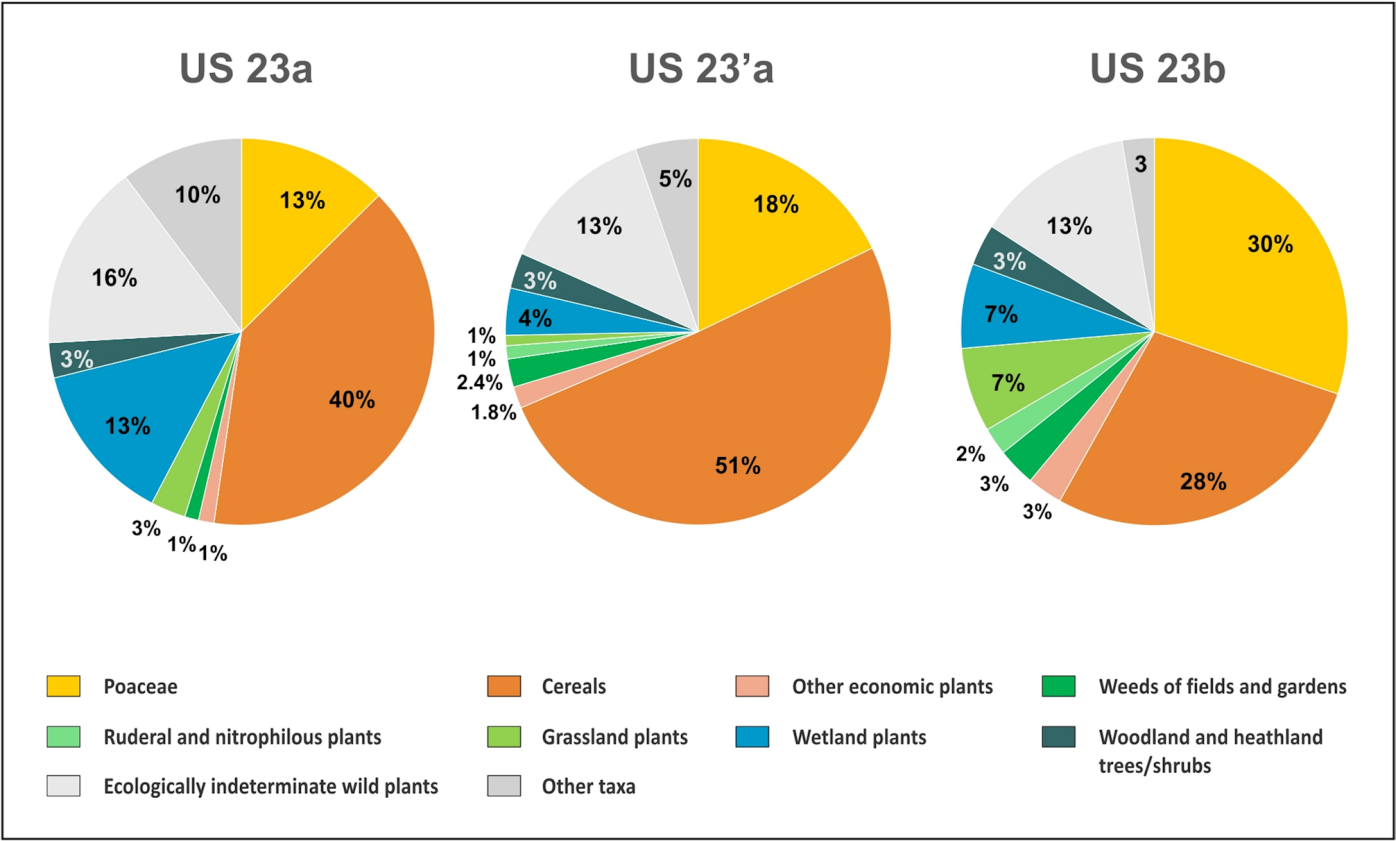
Proportions of the pollen and spores identified in the 3 samples from SU 23 (% of the total pollen sum, NPPs excluded). Samples “**a**” were processed following the first method (ultrasonic sieving), whereas sample “**b**” was treated with the second method (dense media separation). For more details see appendix 1

Figure 11 and supplementary information 2 summarizes the pollen data according to the two different methods applied.

Whereas the traditional protocol only showed a medium to poor preservation of the palynomorphs, a richer spectrum could be observed by applying a more gentle protocol (see methods section). This suggests that, despite the prevailing wet conditions, such archaeological contexts merit the use of the gentle protocol (without ultrasonic sieving).

**Fig. 12:**
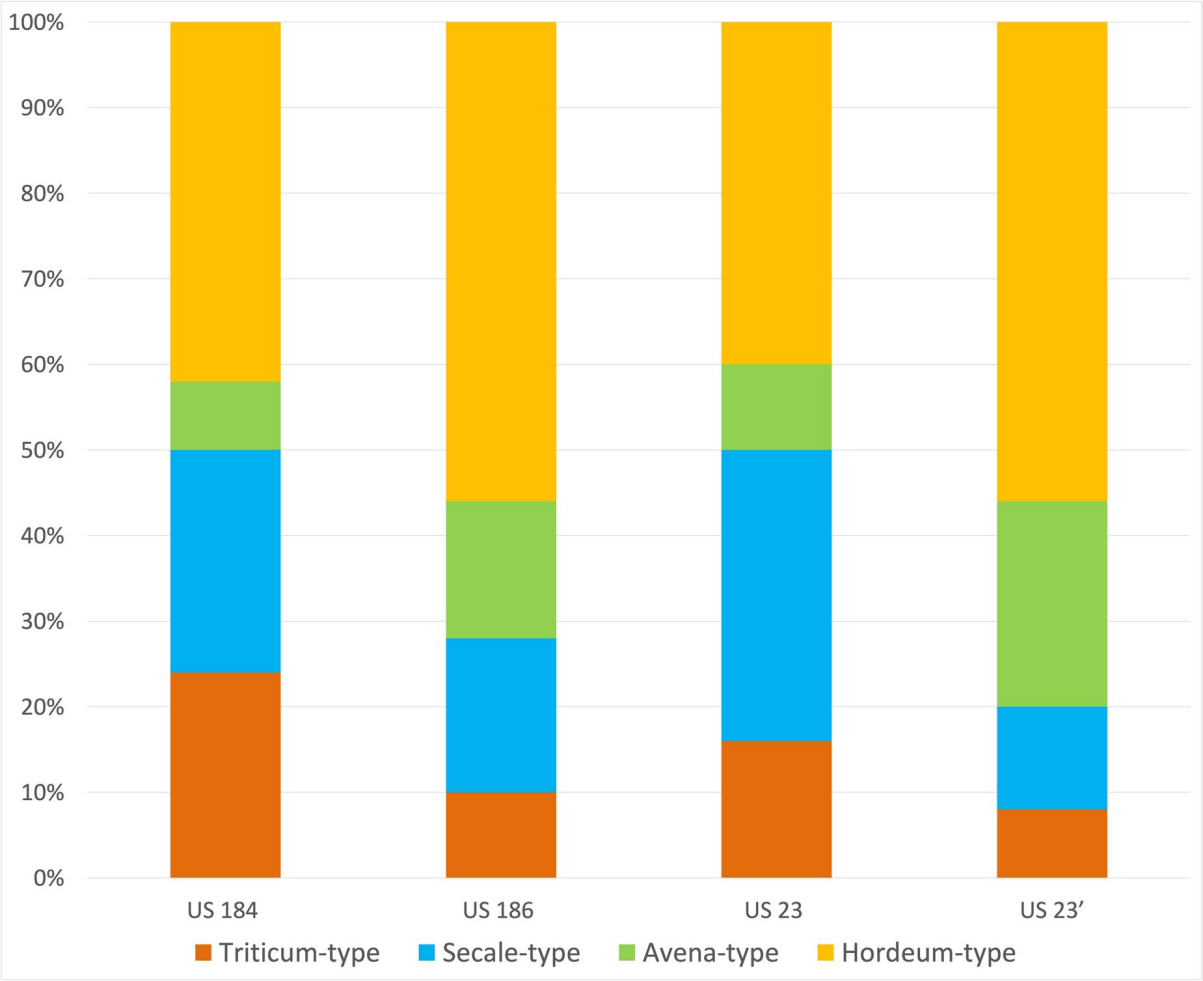
*Cerealia* pollen types identified in percentage proportions. From each sample 50 *Cerealia* pollen grains were taken

Both methods, however, display the same general trends: a clear dominance of the grass and herb pollen, and high frequencies of cultivated cereals. The additional identifications of *Cerealia* pollen (Fig. 12) suggest the dominance *Hordeum*-type, followed by *Avena*- and S*ecale*-type, while the *Triticum*-type pollen is a rather minor component. The other herb vegetation is strongly dominated by pollen of plants growing in disturbed habitats or occurring in the cultivated fields like weeds (*Centaurea cyanus*-type, *Scleranthus annuus*, *Convolvulus* sp., *Mercurialis annua*, *Polygonum aviculare*-type, *Plantago lanceolata*-type, *Agrostemma githago*, *Papaver rhoeas*-type). Most of the tree pollen occurs only sporadically or comes from the local wetland vegetation. Interesting are the finds of cultivated trees like the walnut (*Juglans regia*) and trees, shrubs or lianas which could represent useful or cultivated plants like plum (*Prunus*-type) and hazel (*Corylus avellana*), vine (*Vitis*) and hop/hemp (*Humulus/Cannabis*).

NPPs show the presence of parasitic fungi of cereals, as well as quite large number of coprophilous fungi.

## Discussion

Macroscopically, the fill of the structure seems to be composed of densely packed plant remains, sometimes alternated with more minerogenic and/or humified deposits. The undertaken micro-archaeological and micromorphological studies allow for a detailed characterisation of the structure. Root and mesofaunal galleries are rare, indicating the relatively undisturbed character of the fill. This is supported by the fact that macro- and microstratification is still largely preserved. One example are the articulated systems of phytoliths released from their organic matrix. As in present case, these phytoliths are not glued together as within silica skeletons, they are extremely vulnerable to post-depositional disturbance (see Vrydaghs et al. 2017; Devos and Vrydaghs 2023). The presence of cellulose further suggests an excellent preservation of (part of) the organic remains (Brönnimann et al. 2017a).

Trampling of the deposits is witnessed by the presence of disarticulated phytolith systems, densely packed and broken phytoliths (Fig. 7a). They result of the exertion of physical pressure onto the plant remains in the floor (Devos and Vrydaghs 2023). Further evidence for trampling includes the dominantly horizontally layered plant remains, a phenomenon typical for cases where animals are predominantly fed on grasses (Shahack-Gross 2017), and the strong compaction of the dung fragments. The presence of abundant silt and very fine sand-sized mineral matter in SMT1.2 could result from material attached to the hooves or the fur of the animals or it could also be expelled with urine or faeces by the animals (see Macphail et al. 2004; Macphail and Goldberg 2010; Brönnimann et al. 2017a).

The ubiquitous presence of relatively well-preserved dung fragments suggests that animals were kept for some time inside the structure. The presence of secondary phosphatic nodules and especially the phosphate staining of the herbivorous coprolites suggests that the deposits were probably enriched in urine (Guélat et al. 1998), as herbivorous coprolites are known to be less phosphatic compared to omnivorous/carnivorous dung fragments (see Brönnimann et al. 2017a & b). As the structure is cut within the surrounding dark earth deposits, the identification of a sunken byre or potstal can be advocated. Such a sunken byre results from the repeated cleaning and emptying of the structure. In this respect we can point to SMT2 and SMT2.1 composed of a mixture of aggregates or fragments of vegetal matter and of mineral components without traces of internal layering, probably resulting from material dug from SMT1-like deposits, possibly during the cleaning of animal enclosures.

The lack of textural pedofeatures (e.g. thin films of clay, silt and organic matter that form in the pores due to the splash erosion of raindrops on the surface) might indicate that the byre was located under a protected, hence roofed, environment (see Banerjea et al. 2015a & b). This is also supported by the very limited evidence of mesofaunal and root bioturbation.

The micro-archaeological study indicates that material from different origins has been mixed. The detailed micromorphological observations permit to ascertain at least four different sources.

### Excremental waste and urines

The micromorphological study permitted to identify a series of herbivore excrements, the presence of which is also confirmed by numerous coprophilous fungal spores recorded in the pollen slides. They are typically composed of closely packed plant remains (see Brönnimann et al. 2017a). Beyond providing evidence for the presence of herbivores, these excrements can also provide details on the diet of the animals. For instance, the phytolith study performed on a series of excrements observed within the thin sections demonstrates that the animals were clearly fed with by-products of cereal processing. The content of the herbivore coprolites suggests that the dominant animals kept were non ruminant, more likely horses and/or donkeys. In this respect we point to the omnipresence of rather long streaks of plant remains, typically present in horse/donkey dung (Brönnimann et al. 2017a, see also: Macphail et al. 2004). Further evidence includes the presence of horse- or donkeyhair observed during the micro-archaeological study. Palaeoparasitological observations indicate the potential presence of *Parascaris equorum*. However, the parasitological data should be interpreted with caution as the issue of egg size is yet not resolved. The presence of occasional faecal material containing spherulites may point to the presence of capriovide or cattle dung (see Canti 1997; 1999; Brönnimann 2017a). This observation could also align with the presence of whipworm eggs (*Trichuris* sp.) or the sporadic occurrence of nematode eggs (*Capillaria* sp.), both parasites known to affect capriovide, but also cattle, dogs, cats, birds (e.g. chicken, pigeon) and small rodents (Thienpont et al. 1990).

Although a dominance of herbivore excrements was observed, some carnivorous and omnivorous (possibly human) excrements were present (Fig. 7b). In addition, endoparasite eggs of *Ascaris lumbricoides* and/or *A. suum* are sporadically observed. However, their similar morphology preclude definitive identification of humans or pigs as the primary hosts (Gonçalves et al. 2003; Loreille and Bouchet 2003). This suggests that pigs also had access to the structure at some point, and/or that cess remains were thrown onto the stable floor (see Spek 2004). Small ingestible fruit pips, such as those of *Fragaria vesca*, *Rubus fruticosus*, *Rubus idaeus* and *Vitis vinifera*, are the main elements in plant assemblages from medieval cesspits in the region (e.g. De Cupere et al. 2021). Therefore, these finds (3% of the carpological remains) can be an additional indication for human cess in the stable floor, which would also explain the presence of tapeworm eggs – if they are not from dog, cat or fox (Thienpont et al. 1990).

Intestinal parasites have been a ubiquitous companion of both animals and humans throughout history (Morandi 2018). Common infections, usually due to poor hygienic conditions, often involved whipworms (*Trichuris* sp.) and roundworms (*Ascaris* sp.). In most cases, individuals coexisted with these parasites with minimal health consequences.

### Fodder and bedding material

Evidence for fodder includes the presence of wood remains (including bark and twigs), and a dominance of cereal remains in the pollen and plant macroremains spectra. As already mentioned, the presence of cereal phytoliths within the excrements confirms that cereal fodder was consumed by the animals. Furthermore, the detailed study of the phytoliths within the layered plant remains indicates that part of the cereals seems to be added as bedding material (see higher). This is further confirmed by the clear dominance of cereal chaff and stalk fragments within the plant macroremains and a high proportion of *Cerealia* pollen usually associated with cereal chaff and grains. The straw fragments and *Secale cereale* rachis internodia can be interpreted as by-products of threshing, which were most likely used as stable bedding, but which may partly have been eaten (Derreumaux 2005). *Avena sativa* was commonly used as animal fodder in the region, especially but not only for horses (e.g. Kalkman 2003; Charruadas 2011). The *Avena* florets, still enclosing the grains after harvesting, are likely to be the remains of unhulled oats grains used as fodder. Consequently, these chaff remains can also be interpreted as elements from animal dung. Remains of field and garden weeds and grasslands are very commonly found in stable manure and many species listed by Kenward & Hall (2012) are also found in the stable floor remains from Brussels. Harvested cereals and cereal by-products generally include weeds and it is likely that they were added to the context (partly unintentionally) with cereal fodder and litter. Remains of grassland plants, which are more numerously found and more diverse than in the other studied context at the site (Speleers et al. 2016), can partly be interpreted as elements of hay. Another part of the grassland remains and part of the wetland remains were probably consumed by grazing animals and were deposited on the stable floor as elements of dung.

### Plaggen and/or soil sods

Besides the presence of silt and fine sand mineral matter embedded in the compacted dung matrix (SMT1.2), the micromorphological study also shows the presence of lumps of sandy mineral material (SMT 3.2) probably deliberately imported in the sunken byre. Additionally, some lumps may originate from soil sods. This could explain the presence of wetland vegetation, heather pollen and plant macroremains. Either these sods have been added to raise the floor level within this wet environment – although it is rather difficult to explain why they would use soil from wetlands to achieve this -, or they have been added to the stable to allow the animals to enrich the sods with urines and excrements. Indeed, ethnographic and historical studies demonstrate that farmers regularly added soil sods from wetlands and heathlands to sunken byres (see Domhof 1953; Lindemans 1994; Spek 2004). Besides, the presence of diatoms, chrysophycean stomatocysts, sponge spicules fits well with the valley position of the site, located therefore in a wet environment. Such microfossils can be trampled in by animals or people, can be imported with sediments, can be ingested with drinking water and expelled with faeces/urines, or can indicate the use of plant material growing in neighboring areas as fodder or bedding (e.g. sedges). In the study of a Roman sunken byre in the site of Brecht—Zoegweg (provincie Antwerp – see Mikkelsen et al. 2019), the presence of diatoms and iron nodules was interpreted as a proof of the import of plant matter and attached soil material from the nearby acidic heathland. This land management model fits with the ethno-historical models found in Spek (2004).

### Household and construction waste

Evidence for household waste includes the presence of charcoal (mainly from beech and to a lesser extent of oak). These result probably from residues of fire wood as beech was preferred fuel wood or its ashes were used in glass making (Gale and Cutler 2000). Rare calcareous ashes were observed. Originally, these ashes must have made up a more important part of the structure, but due to the acidic conditions, most of it was not preserved. Further components are ceramics and bone fragments. Part of the carpological remains, such as the larger fruit stones, may also be interpreted as household waste. According to Spek (2004), domestic waste, and even cess (see higher) was often added to the potstal.

Some dense fragments of mineral material were also observed, possibly pointing to the presence of microscopic fragments of earthen-based construction material (see Macphail and Devos 2023). Again, these may have entered the structure either through trampling or they may result from the degradation of the walls of the structure.

The different origins of the content of the potstal can explain the different taphonomy of the pollen and plant macroremains, thus when combined widen the evidence on the plant economy (see Vandorpe and Wick 2015).

The herbal vegetation as recorded in the pollen spectra, apart of grasses is strongly dominated by pollen of plants growing in disturbed habitats or occurring in the cultivated fields like weeds (*Centaurea cyanus*-type, *Scleranthus annuus*, *Polygonum aviculare*-type, *Convolvulus*, *Mercurialis annua*). They can be associated with threshing remains used as fodder and stable floor covering. The subfossil wood could originate from local vegetation or activities related with foddering. Especially, the presence of small diameter twigs including such of hazel in some of the samples could reflect the winter foddering with foliage, a quite plausible practice for this location.

## Archaeological/historical implications

Sunken byres are a common phenomenon from Roman times onwards in the Belgian Campine area (see Mikkelsen et al. 2019). They were an important component in the farming system, as they provided organic and nutrient rich manure to fertilize the poor sandy soils (Lindemans 1994). So far, no such structures have been observed in Middle Belgium, an area dominated by loess deposits. However, taking a close look at the area, we can observe that along the steep slope leading from the Senne valley to the Brabantian plateau, the loess cover is very thin or even absent. As a result, this area is dominated by poor sandy topsoils that require a continuous and substantial amount of organic and nutrient rich manure. This may explain the occurrence of this kind of stable. Moreover, historians claim the importance of an agro-pastoral system where animals were kept in stables over winter, as part of the explanation for cultivating new and less rich soils in high medieval times (see Charruadas 2011). The intensive study of high medieval cultivated fields in the historical centre of Brussels indeed shows that they have intensively been amended with (stable) manure (see Devos et al. 2007; 2011a; 2011b; 2013; 2017; Devos 2019).

## Conclusions

The interdisciplinary study of the byre remains allows to detail the content of the fill, its origins and to understand the processes involved. It provides relatively detailed insights into the foddering customs, hygienic conditions within the stable, and the health status of the animals kept. On a more general scale this study allows to get a better idea on late medieval farming practices in Brussels, more specifically the need to collect substantial amounts of manure to add as fertilizer onto the cultivated poor sandy soils.

## Supplementary Information

Below is the link to the electronic supplementary material.

Supplementary file1 (PDF 65 KB)

Supplementary file2 (PDF 70.3 KB)

## Data Availability

Supporting data can be found within the manuscript and the supplementary information.
