# Supplementary material for: Understanding late medieval farming practices: an interdisciplinary study on byre remains from the historical centre of Brussels (Belgium)
Source: Archaeol Anthropol Sci. 2025 Jun 25;17(7):154. doi: 10.1007/s12520-025-02248-w (PMC12198283; doi:10.1007/s12520-025-02248-w)
Supplement: Supplementary file 1 — Supplementary file1 (PDF 65 KB) [file 12520_2025_2248_MOESM1_ESM.pdf]

Period	13th c.	13th c.	13th c.
Stratigraphic unit	US 23a	US 23’a	US 23b
**Pollen sum**	**1463**	**1269**	**782**
**Arboreal pollen (sum)**	**107**	**69**	**57**
Pinus sylvestris-type	14	13	6
Juniperus communis-type	0	1	13
Fagus sylvatica	2	10	2
Quercus robur/pubescens-type	6	2	0
Tilia	18	6	0
Ulmus	3	0	0
Corylus avellana	3	15	5
Betula	11	0	6
Alnus glutinosa/incana	28	8	1
Salix	6	1	0
Sambucus nigra-type	0	2	0
Prunus-type	14	3	15
Juglans regia	2	0	1
Humulus/Cannabis	0	2	0
Vitis	0	0	2
Ericaceae	0	6	1
Calluna vulgaris	0	0	5
**Non-Arboreal pollen (sum)**	**473**	**449**	**438**
Cerealia	581	643	218
Linum usitatissimum	0	1	0
Cenraurea cyanus-type	2	5	8
Scleranthus annus	6	0	0
Polygonum aviculare-type	0	13	5
Convolvulus	2	0	2
Mercurialis annua	0	0	1
Anthemis-type	2	6	5
Vicia-type	5	6	4
Poaceae	184	227	236
Chenopodiaceae	144	133	41
Brassicaceae	43	22	14
Apiacaeae	11	0	12
Caryophyllaceae	8	3	0
Cichorioideae	29	0	32
Aster-type	26	5	28
Centaurea nigra-type	0	0	2
Plantago lanceolata-type	9	0	11
Plantago sp.	0	2	0
Galium-type	2	2	3
Ranunculus acris-type	0	0	12
Scrophularia-type	0	9	1
Rhinanthus type	0	0	1
Helianthemum	0	0	1
Alchemilla	0	1	0
Urtica-type	0	10	0
Artemisia	0	0	9
Rumex acetosa-type	0	3	6
Lamium album-type	0	0	3
Carduus-type	0	1	0
**Wetlands/aquatics**	**152**	**41**	**48**
Persicaria aquatica	64	38	0
Mentha-type	2	0	6
Lythrum salicaria	0	3	0
Filipendula	0	0	2
Solanum dulcamara	0	0	1
Cyperaceae	86	0	39
**Ferns**	**94**	**0**	**11**

Equisetum	69	0	2
Polypodium vulgare	25	0	0
Trilete spores	0	0	7
Riccia sorocarpa	0	0	2
Indet. (corroded)	44	53	0
Indet.	12	14	10
**Non-Pollen-Palynomorphs (excluded from the pollen sum)**			
Sordaria sp. (fungi)	21	24	38
Chaetomium sp. (fungi)	0	10	45
Podospora sp. (fungi)	0	0	10
Arnium sp. (fungi)	0	0	10
Zopfiella sp. (fungi)	0	0	10
Ustilaginales Type 710/728 (fungi)	0	0	9
Tripterospora sp. (fungi)	0	0	7
Thecaphora sp. (fungi)	0	0	6
Trichodelitschia sp. (fungi)	0	0	5
Glomus fasciculatum (fungi)	0	0	1
Type 203/206 (fungi)	0	0	8
Type 708 (fungi)	0	0	32
Mycelium (fungi)	0	0	18
Chrysophyceae (algae)	0	0	16
Pseudoschizaea sp. (algae ?)	0	0	1
Eurycercus lamellatus (aquatic invertebrate)	0	0	1
Trichuris sp. (parasite egg)	0	0	24
Betula (trachea)	0	0	1
