# Supplementary material for: Understanding late medieval farming practices: an interdisciplinary study on byre remains from the historical centre of Brussels (Belgium)
Source: Archaeol Anthropol Sci. 2025 Jun 25;17(7):154. doi: 10.1007/s12520-025-02248-w (PMC12198283; doi:10.1007/s12520-025-02248-w)
Supplement: Supplementary file 2 — Supplementary file2 (PDF 70.3 KB) [file 12520_2025_2248_MOESM2_ESM.pdf]

Understanding late medieval farming practices: an interdisciplinary study on byre remains from the historical centre of Brussels (Belgium)

Yannick Devos[1], Cristiano Nicosia[2], Luc Vrydaghs[1], Jan M. A. van der Valk[3], Lien Speleers[6], Elena Marinova[4,6], Mona Court-Picon[6], Terry B. Ball[7], Christine Pümpin[8], Hugues Doutrelepont † [5], Britt Claes[9]

1: Archaeology, Environmental Changes & Geo-Chemistry Research Group, Vrije Universiteit Brussel, Brussels, Belgium
2: Università di Padova, Padua, Italy
3: Independent researcher, Belgium
4: Baden-Württemberg State Office for Cultural Heritage, Hemmenhofen, Germany
5: Roots asbl, Brussels, Belgium
6: Royal Belgian Institute of Natural Sciences, Brussels, Belgium
7: Brigham Young University, USA
8: IPNA, University of Basel, Basel, Switzerland
9: Royal Museums for Art and History, Brussels, Belgium

Corresponding author: Yannick Devos, Yannick.george.devos@vub.be

Supplementary Material 1: Soil micromorphological descriptions

SMT 1 is characterized by a crenulated aspect and a platy microstructure. The unit is closely packed. Pores are mainly planar voids, channels and pseudomorph voids. The $c/f_{(5\mu m)}$ related distribution is coarse monic to dominantly close porphyric. The few coarse mineral fraction is essentially composed of silt to coarse sand sized quartz. The main additional minerals are glauconite and muscovite. The fine fraction is dark brown to black and isotropic, locally crystallitic. The organic material consists mostly of parallel layers (lenses) of brownified to blackened organic remains. Plant cells, parenchymatic tissues (containing cellulose), dicotyledon wood organs (twigs) and lignified brown tissues (probably leaves), seed coat fragments and (fungal) spores and more humified plant remains are observed. We further noted the presence of endoparasite eggs. Isolated, clustered and articulated phytoliths were noted, as well as some clusters of faecal spherulites, isolated diatoms and chrysophycean cysts. The articulated phytoliths were often still incorporated within the organic tissues. Very few charcoal and mortar fragments are observed. Locally pyrite framboids are noted.

SMT 1.1 is composed of compressed dung and presents a dominantly apedal microstructure. Pores are mainly planar voids. The $c/f_{(5\mu m)}$ related distribution is open porphyric. The few coarse mineral fraction is essentially composed of few silt to coarse sand sized quartz. The main additional mineral is glauconite. The fine fraction is orange brown, isotropic and auto-fluorescent phosphate. The organic material consists of horizontal compacted parallel lenses of blackened and ferruginized organic remains. Plant cells, phlobaphene containing tissues, parenchymatic tissues (rich in cellulose), lignified brown tissues, wood organs, pollen, (fungal) spores and seed coats are present. We further noted the presence of endoparasite eggs. Isolated, clustered and articulated phytoliths are omnipresent. Isolated diatoms are also noted.

SMT 1.2 is characterized by its crenulated aspect and presents a massive to platy microstructure. Pores are mainly planar voids and pseudomorphs. The $c/f_{(5\mu m)}$ related distribution is open to double spaced porphyric. The coarse mineral fraction is essentially composed of moderately to well sorted silt to very fine sand, with few medium sand sized quartz. The main additional minerals are glauconite and muscovite. The micromass is dark brown, organic, isotropic with undifferentiated b-fabric. The organic material is besides the fine organic material composed of elongated vegetal tissue/organ fragments, horizontally layered with close packing, dominant. Scarce twigs, roots and wood are observed. Endoparasite eggs are also reported. Isolated, clustered and articulated phytoliths are abundantly present. Diatoms, sponge spicules and chrysophycean cysts are noted. Very scarce mortar, scarce charcoal, rare volcanic rock fragments (potentially fragments of millstones) and carnivore/omnivore coprolite fragments are observed.

SMT 1.3 presents a massive microstructure. Porosity is low and mainly composed of rare vughs. The $c/f_{(5\mu m)}$ related distribution is open porphyric. The coarse mineral fraction is essentially composed of well sorted silt to fine sand sized quartz. The main additional minerals are glauconite and muscovite. The micromass is dark brown, organic, isotropic with undifferentiated b-fabric. The organic material is, besides the dark brown fine organic material, composed of vegetal tissue and organ fragments, including wood. Poorly visible isolated, clustered and articulated phytoliths, diatoms, sponge spicules and chrysophycean cysts were noted.

SMT 2 presents a granular to crumby microstructure. Pores are mainly channels, vughs, planar and packing voids. The $c/f_{(5\mu m)}$ related distribution is chito-enaulic. The coarse fraction is composed of well sorted silt to very fine sand sized quartz (< 10 coarse sand sized quartz is also present). The main additional mineral are feldspar, glauconite and muscovite. The micromass is black to dark brown and poorly birefringent. The organic material is randomly distributed and composed of unoriented blackened organic remains. Plant cells, parenchymatic tissues (rich in cellulose) and lignified brown tissues (probably leaves), seed coat fragments, stone pits and (fungal) spores and hyphae are noted, as well as endoparasite eggs. Isolated, clustered and articulated phytoliths are observed, as well as diatoms, sponge spicules and chrysophycean cysts. Anthropogenic elements include charcoal, mortar fragments, eggshell, shell, scarce burned bone, rare volcanic rock fragments (potentially fragments of millstones) and dense rounded soil fragments. Herbivore and omnivore/carnivore coprolites have been noticed. Phosphatic crystal intergrowths and locally rare vivianite nodules are observed. Locally pyrite framboids are noted.

SMT 2.1 presents a very porous crumby microstructure. Pores are mainly channels, vughs, planar voids and packing voids. The $c/f_{(5\mu m)}$ related distribution is open porphyric. The coarse mineral fraction is less abundant compared to SMT 2 and is composed of moderately sorted and mainly composed of silt to medium sand sized quartz. Main additional minerals are glauconite and muscovite. The micromass is dotted, dark brown, isotropic and iron stained. The organic material is dominated by fragmented unoriented humified organic remains. Cells, phlobaphene containing tissue, parenchymatic tissue, lignified tissue, needles, wood fragments and seed coats are also present, as well as

endoparasite eggs. Rare carnivore/omnivore and herbivore excrements are noted. Isolated, clustered and articulated phytoliths are observed, as well as diatoms, sponge spicules and chrysophycean cysts. Anthropogenic elements include charcoal, bone and burned bone, burned eggshell, mortar and reworked dense soil fragments. Vivianite crystal intergrowths are observed.

SMT 3 presents a channel microstructure. Pores include channels and pseudomorphic voids. The $c/f_{(5\mu m)}$ related distribution is close porphyric. The coarse mineral fraction is moderately sorted and composed of silt to medium sand sized quartz. Additional minerals are glauconite and muscovite. The fine material is dotted, dark brown, organic, poorly birefringent to isotropic, undifferentiated b-fabric. The organic material is composed of roots, vegetal tissue fragments, scarce wood fragments and organic punctuations. Isolated and clustered phytoliths are observed, as well as diatoms and chrysophycean cysts. Charcoal and charred vegetal matter is noted (ca. 20% at 100x magnification). Pedofeatures include vivianite crystal intergrowths.

SMT 3.2 presents a massive microstructure. Pores are mainly planar voids. The $c/f_{(5\mu m)}$ related distribution is close porphyric. The coarse mineral fraction is well sorted and composed of silt to very fine sand quartz. Additional minerals are glauconite and muscovite. The fine material is light gray, isotropic. The organic material is limited to scarce blackish organic punctuations. Isolated and clustered phytoliths are observed, as well as chrysophycean cysts. Traces of clay illuviation (limpid clay coatings) are present.
